# Longitudinal trajectories of severe wheeze exacerbations from infancy to school age and their association with early‐life risk factors and late asthma outcomes

**DOI:** 10.1111/cea.13553

**Published:** 2020-01-21

**Authors:** Matea Deliu, Sara Fontanella, Sadia Haider, Matthew Sperrin, Nophar Geifman, Clare Murray, Angela Simpson, Adnan Custovic

**Affiliations:** ^1^ Division of Informatics, Imaging, and Data Science Faculty of Medicine, Biology, and Health University of Manchester Manchester UK; ^2^ National Heart and Lung Institute Imperial College of Science Technology, and Medicine London UK; ^3^ Division of Infection, Immunity, and Respiratory Medicine School of Biological Sciences University of Manchester Manchester UK

**Keywords:** asthma exacerbations, childhood asthma, machine learning, primary care data

## Abstract

**Introduction:**

Exacerbation‐prone asthma subtype has been reported in studies using data‐driven methodologies. However, patterns of severe exacerbations have not been studied.

**Objective:**

To investigate longitudinal trajectories of severe wheeze exacerbations from infancy to school age.

**Methods:**

We applied longitudinal k‐means clustering to derive exacerbation trajectories among 887 participants from a population‐based birth cohort with severe wheeze exacerbations confirmed in healthcare records. We examined early‐life risk factors of the derived trajectories, and their asthma‐related outcomes and lung function in adolescence.

**Results:**

498/887 children (56%) had physician‐confirmed wheeze by age 8 years, of whom 160 had at least one severe exacerbation. A two‐cluster model provided the optimal solution for severe exacerbation trajectories among these 160 children: “Infrequent exacerbations (IE)” (n = 150, 93.7%) and “Early‐onset frequent exacerbations (FE)” (n = 10, 6.3%). Shorter duration of breastfeeding was the strongest early‐life risk factor for FE (weeks, median [IQR]: FE, 0 [0‐1.75] *vs.* IE, 6 [0‐20], *P* < .001). Specific airway resistance (sR_aw_) was significantly higher in FE compared with IE trajectory throughout childhood. We then compared children in the two exacerbation trajectories with those who have never wheezed (NW, n = 389) or have wheezed but had no severe exacerbations (WNE, n = 338). At age 8 years, FEV_1_/FVC was significantly lower and FeNO significantly higher among FE children compared with all other groups. By adolescence (age 16), subjects in FE trajectory were significantly more likely to have current asthma (67% FE *vs.* 30% IE *vs.* 13% WNE, *P* < .001) and use inhaled corticosteroids (77% FE vs. 15% IE *vs.* 18% WNE, *P* < .001). Lung function was significantly diminished in the FE trajectory (FEV_1_/FVC, mean [95%CI]: 89.9% [89.3‐90.5] *vs.* 88.1% [87.3‐88.8] *vs.* 85.1% [83.4‐86.7] *vs.* 74.7% [61.5‐87.8], NW, WNE, IE, FE respectively, *P* < .001).

**Conclusion:**

We have identified two distinct trajectories of severe exacerbations during childhood with different early‐life risk factors and asthma‐related outcomes in adolescence.

## 1 INTRODUCTION

Asthma is the most common chronic disease in children. Despite advances in treatment and changes in guidelines, severe exacerbations continue to occur, thus creating a major burden on not only the child and the child's family, but also on healthcare resources.^1^ Notably, a small proportion of children with frequent severe exacerbations account for the majority of the total disease burden in childhood.^2^ Recent studies utilizing data‐driven methods have shown that exacerbations may provide meaningful insight into understanding asthma heterogeneity.^3^, ^4^ Identified risk factors for severe exacerbations include younger age, male sex, race, parental smoking history, socio‐economic status, diminished lung function, severe exacerbation in previous year, respiratory virus infections, and synergistic effects of allergen sensitization, exposure and virus infection.^5^, ^6^, ^7^, ^8^, ^9^, ^10^, ^11^ However, despite the current advancement of knowledge and treatment options in this domain, preventing an impending exacerbation remains difficult.^12^ A recent randomized controlled trial in school‐age children has shown that quintupling the dose of inhaled corticosteroids (ICS) at the early signs of loss of asthma control did not prevent a full‐blown exacerbation.^13^

Exacerbations encompass the crux of the definition of severe asthma, in that asthma is considered to be severe if a patient had frequent severe (use of oral steroids) or serious (hospital admission) exacerbations along with poor symptom control and airflow limitation.^14^, ^15^ However, children can have asthma attacks despite good adherence with treatment and relatively good symptom control. Studies using data‐driven methods have shown that exacerbations are present within different asthma clusters of varying disease severity,^3^, ^4^ and that patients with mild disease can have high rates of severe exacerbations, whereby decreasing symptoms may not always mean a decrease in the number of exacerbations.^7^, ^16^ Using data‐driven techniques, we have recently identified an exacerbation‐prone asthma cluster that contained a proportion of children with mild asthma and normal lung function.^3^ This data suggests that patients with exacerbations may account for a separate susceptibility phenotype, relatively independent of whether or not a child is deemed to have severe asthma, with a likely unique aetiology.

We hypothesized that there are distinct patterns of severe exacerbations of wheezing/asthma during childhood, and that uncovering such patterns may help ascertain whether they are underpinned by different mechanisms. To address our hypothesis, within an unselected birth cohort, we identified longitudinal trajectories of severe exacerbations of wheezing by analysing exacerbation patterns from birth to school age. We then examined their early‐life risk factors, and asthma‐related outcomes and lung function measures in late childhood.

## 2 METHODS

### 2.1 Study population

The Manchester Asthma and Allergy Study is a population‐based birth cohort and is described in detail elsewhere.^17^ Subjects were recruited prenatally and followed prospectively. The study was approved by the Local Ethics Committee. Parents provided written informed consent. For more details about methods and variable definitions please see Online supplement.

### 2.2 Data sources and definition of variables to identify exacerbation trajectories

We extracted data from electronic and paper‐based primary care medical records, including prescriptions of any medication (type, dose and indication) including oral corticosteroid, episodes of wheeze, emergency department admissions, and asthma or wheeze‐related admissions to hospital. Age in days at the time of each event was documented.^18^ These data were available from birth to age 8 years.

#### 2.2.1 Severe exacerbation of wheeze/asthma

Defined from medical records as either receipt of oral corticosteroids (OCS) for at least 3 days, or emergency department visit because of asthma/wheeze requiring systemic corticosteroids or hospital admission.^19^ We ascertained age in days of each exacerbation to provide an accurate account of each episode.

### 2.3 Data sources and definition of variables to validate exacerbation trajectories

Children attended clinical follow‐ups at ages 1, 3, 5, 8, 11 and 16 years. Validated questionnaires were interviewer‐administered to collect information on symptoms, environmental exposure and treatments received. Early‐life risk factors, including environmental tobacco smoke (ETS) exposure, pet ownership, length of breastfeeding, day‐care attendance, presence of older siblings and family history of asthma, were ascertained during the last trimester of pregnancy and in the first year of life. Allergic sensitization was determined using skin prick tests (SPT) to common inhalant and food allergens.

#### 2.3.1 Current asthma at age 16 years

Defined as the presence of any two of the following three features: 1) Current wheeze; 2) Current use of asthma medication; and 3) Physician‐diagnosed asthma ever.^20^

#### 2.3.2 Asthma severity

We created a composite variable to represent symptom control and step in medication based on the British Thoracic Society (BTS) guidelines.^21^ Children on BTS step 1 and 2 were considered to have ‘Mild asthma’, and those on steps 3 and above ‘Moderately severe asthma’. Missing data were considered to be missing at random.

#### 2.3.3 Lung function, airway hyperreactivity and airway inflammation

We measured lung function using spirometry at ages 8, 11 and 16 years using a Lilly pneumotachograph with animated incentive software (Jaeger, Würzburg, Germany), or for home visits, a flow turbine spirometer (Micro Medical, UK).^22^ FEV_1_% predicted^23^ and FEV_1_/FVC ratio were recorded.

Specific airway resistance (sR_aw_) was measured using plethysmography (Masterscreen Body 4.34; Jaeger) at ages 3, 5, 8, 11 and 16.

Airway hyperreactivity (AHR) was measured using standard protocol with quadrupling doses of methacholine in a 5‐stage process at age 8.^24^ We calculated dose‐response slope^25^ as indicator of AHR.

We measured Fractional Exhaled nitric oxide (FeNO) as a biomarker of airway inflammation at ages 8 and 16 years according to the American Thoracic Society guidelines using either a chemiluminescence analyser or an electrochemical analyser (NIOX, Solna, Sweden).^26^ Data were expressed in parts per billion (ppb).

### 2.4 Statistical Analysis

#### 2.4.1 Exploratory analysis to identify trajectories of exacerbations

For ascertaining exacerbation patterns, we used primary care records data from birth to age 8 years, as this provided an objective account of hospital admissions, receipt of systemic corticosteroids and presence of wheeze. We used cluster analysis for longitudinal data to identify whether there are subgroups of patients with similar patterns of severe exacerbations. We applied a longitudinal extension of the k‐means algorithm (KmL, a partitional clustering method),^27^ and ascertained the optimal number of clusters using the Calinski‐Harabatz criterion.^27^ Results for *post hoc* longitudinal cluster analysis were obtained through the KmL package developed in R software.^28^, ^29^ Technical details are provided in the online supplement.

#### 2.4.2 Characteristics of different trajectories of severe exacerbation

To assess differences between clusters (trajectories), we used either a t test, χ^2^ test, Mann‐Whitney test or one‐way ANOVA as appropriate. To check which early‐life risk factors predict trajectory membership, we used logistic regression models.

In order to identify differences between children with different exacerbation trajectories, those with wheezing but no exacerbations, and children who have never wheezed, we used multinomial logistic regression models with lung function, AHR, allergic sensitization, asthma severity and asthma medication as outcomes. All analysis was performed using R software (http://www.r-project.org/).^30^

## 3 RESULTS

### 3.1 Population characteristics and participant flow

Of 1184 children born into the cohort, 984 families gave consent for the review of medical records, of whom we extracted data for 887 children. Of those, 498 (56%) had physician‐confirmed wheeze in their medical records on at least one occasion up to age 8 years, and 389 never wheezed. Of 498 children with wheezing, 338 had no exacerbations, and 160 (32%) had at least one confirmed severe exacerbation in the first 8 years; total number of exacerbations among these 160 children was 271. The number of children with at least one severe exacerbation in each year of life from birth to age 8 years is shown in Table E1: the annual incidence was much higher in the first 4 years of life (5%), and then steadily reduced between ages 4 and 8 years to <2%. The median age of the first confirmed wheeze episode was 676 days (IQR: 187‐863), and of the first severe exacerbation 893 days (IQR: 343‐1238).

Descriptive characteristics of 160 children who had at least one severe exacerbation of wheezing are shown in Table E1; 68 (43%) had a physician diagnosis of asthma in the same period, and 116 (73%) were prescribed ICS at some point. Current use of ICS among current exacerbators in each 12‐month period from birth to age 8 increased from 31% in the first year of life to 79% in year 8. Among children with exacerbation(s), on average 11% per annum had ≥ 3 exacerbations in the preceding 12 months; all but one of these children received asthma medication.

### 3.2 Identification of trajectories of severe exacerbations and their associates

**Figure 1:**
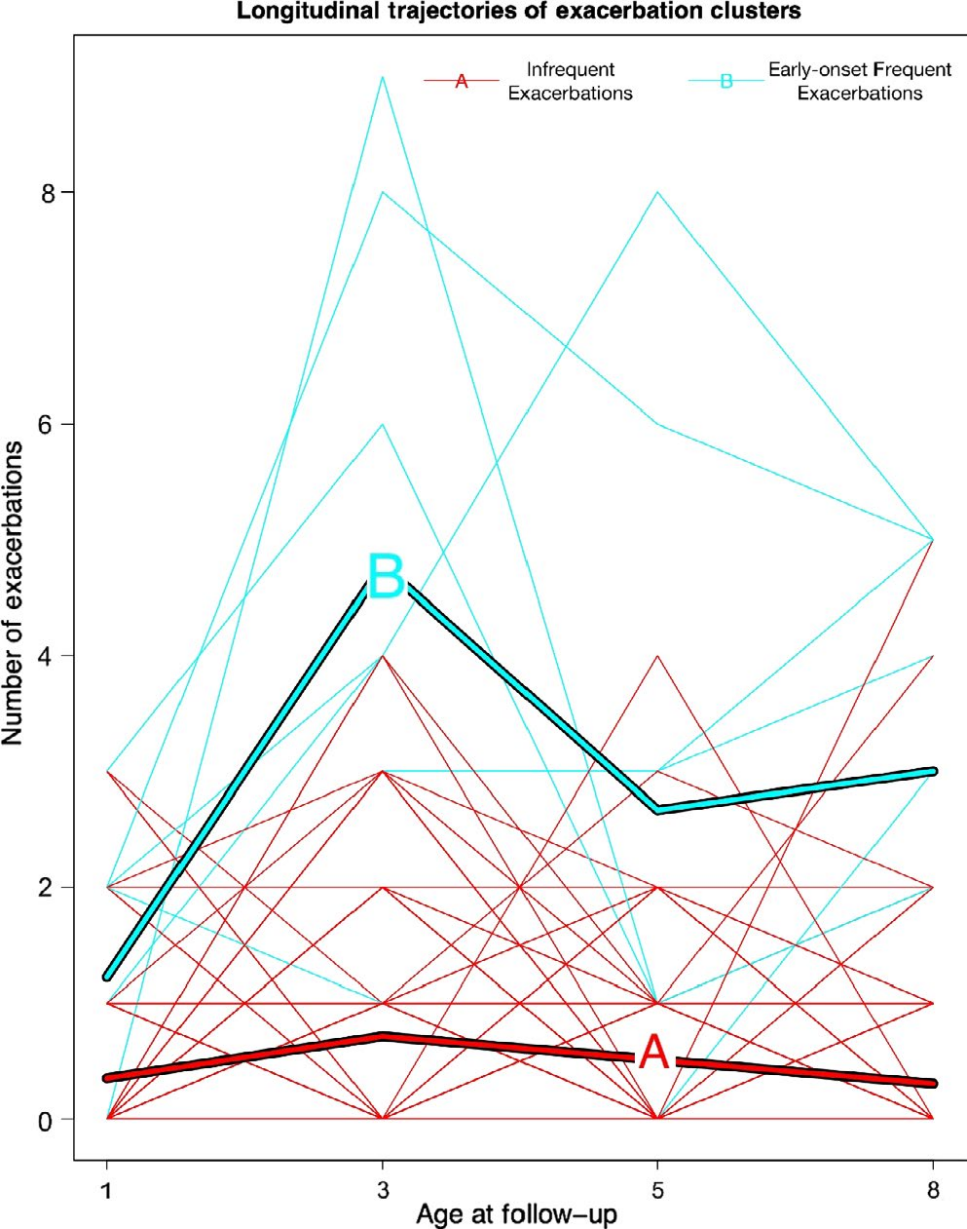
Longitudinal trajectories of exacerbations. Cluster A: infrequent exacerbations, N = 150 (93.7%). Cluster B: Early‐onset frequent exacerbations, N = 10 (6.3%). Each line represents an individual pattern of exacerbations within each trajectory

To identify exacerbation trajectories, we analysed data from 160 children who had at least one confirmed severe exacerbation from birth to age 8 years. According to the Calinski‐Harabatz index (Figure E1), the optimal model that best described the data was a 2‐class solution. Figure 1 shows the exacerbation patterns in the two clusters (trajectories), and individual pattern of exacerbations within each trajectory. We assigned trajectories as “Infrequent exacerbations (IE)” (n = 150 [93.7%], median number of exacerbations = 1) and “Early‐onset frequent exacerbations (FE)” (n = 10 [6.3%], median exacerbations number = 4).

#### 3.2.1 Early‐life characteristics and clinical features

The associations of exacerbation trajectories with risk factors and clinical features in the first 8 years of life are presented in Table 1. Shorter duration of breastfeeding was a strong risk factor for FE (median weeks, FE 0 [IQR: 0‐1.75] *vs.* IE 6 (IQR: 0‐20), *P* < .001). Family history of asthma and tobacco smoke exposure did not differ between clusters. Children in FE cluster were significantly more likely to have eczema in the first 3 years of life, but not thereafter. Allergic sensitization and co‐morbid rhinitis did not significantly differ between the clusters. By age 8 years, significantly higher proportion of children in the FE cluster had doctor‐diagnosed asthma (90% *vs.* 39%, *P* = .002). The use of ICS was higher among children in FE cluster, particularly at age 3 years (80% *vs.* 22%, FE *vs.* IE, *P* < .001), and children in this cluster were more likely to have persistent wheeze (90% *vs.* 47%, *P* = .03).

**Table 1:** Early‐life characteristics and clinical features of exacerbation clusters

	Cluster 1 (IF) n = 150	Cluster 2 (FE) n = 10	*P*‐value
Gender (boys)	103 (67%)	5 (50%)	.38
Family history of asthma	51 (34%)	3 (30%)	.86
Younger sibling	48 (32%)	3 (30%)	.84
Older sibling	93 (62%)	5 (50%)	.59
Breastfeeding (weeks), median (IQR)*	**6 (0‐20)**	**0 (0‐1.75)**	**<.001**
Day‐care attendance	78 (52%)	7 (70%)	.36
Tobacco exposure, birth	53 (35%)	5 (50%)	.64
Tobacco exposure 1y	46 (30%)	4 (40%)	.80
Tobacco exposure 3y	46 (30%)	4 (40%)	.88
Tobacco exposure 5y	42 (28%)	5 (50%)	.31
Dog ownership, birth	20 (13%)	3 (30%)	.29
Cat ownership, birth	24 (16%)	4 (40%)	.34
Allergic sensitization (SPT), age 1 year	11 (7%)	2 (20%)	.18
Allergic sensitization (SPT), age 3 years	50 (33%)	5 (50%)	.27
Allergic sensitization (SPT), age 5 years	67 (45%)	4 (40%)	.94
Allergic sensitization (SPT), age 8 years	69 (46%)	6 (60%)	.83
Rhinitis, age 5 years	55 (37%)	5 (50%)	.36
Rhinitis, age 8 years	51 (34%)	6 (60%)	.13
Eczema, age 1 years	**54 (36%)**	**7 (70%)**	**.03**
Eczema, age 3 years	**47 (31%)**	**6 (60%)**	**.03**
Eczema, age 5 years	56 (37%)	4 (40%)	.54
Eczema, age 8 years	48 (32%)	5 (50%)	.24
Doctor‐diagnosed asthma ever	**59 (39%)**	**9 (90%)**	**.002**
Use of inhaled corticosteroids, age 3 years	**33 (22%)**	**8 (80%)**	**<.001**
Use of inhaled corticosteroids, age 5 years	46 (31%)	6 (60%)	.11
Use of inhaled corticosteroids age 8 years	41 (27%)	5 (50%)	.13
Wheeze phenotypes			
Transient early wheeze	15 (10%)	0 (0%)	.84
Late onset wheeze	50 (33%)	1 (10%)	.98
Persistent wheeze	**70 (47%)**	**9 (90%)**	**.03**

Bold values relate to statistically significant results.

Abbreviations: IF, Infrequent exacerbations; FE, Early‐onset frequent exacerbations; SPT, skin prick test, Quantitative variable presented as median (IQR), Ordinal variables represented as frequencies (%).

* Mann‐Whitney test for medians. Chi‐squared for binary variables.

The distribution of asthma severity in two exacerbation clusters is shown in Table E2. Although most children in both clusters had mild asthma, those in FE cluster accounted for the higher percentage of more severe asthma, with significant difference between clusters at age 3 years (*P* = .03).

Table 2 shows lung function, airway inflammation and AHR among children in the two exacerbation clusters. sR_aw_ was markedly and significantly higher in FE compared with IE throughout childhood (Figure E2). At age 8 years, children in the FE trajectory had significantly lower FEV_1_ and FEV_1_/FVC (mean [95% CI]: FEV_1_% predicted, 95.6 [93.3‐97.9] *vs.* 91.1 [80.9‐101.3], *P* < .001; FEV_1_/FVC 85.1% [83.9‐86.2] *vs.* 78.1% [72.8‐83.4], *P* < .001), and significantly higher FeNO (ppb, mean [95% CI] 11.5 [7.8‐19.5] *vs.* 58.5 [24.2‐79.3], *P* < .001). AHR was higher in FE cluster, but this did not reach statistical significance (*P* = .08).

**Table 2:** Lung function, non‐specific airway reactivity and airway inflammation among children with infrequent exacerbations (IF) and those with early‐onset frequent severe exacerbations (FE)

	Infrequent Exacerbations (IF, n = 150) mean (95%CI)	Early‐onset Frequent Exacerbations (FE, n = 10) mean (95%CI)	*P*‐value
FEV_1_% predicted, age 8	95.6 (93.3‐97.9)	91.1 (80.9‐101.3)	**<.001**
FEV_1_/FVC, age 8	85.1 (83.9‐86.2)	78.1 (72.8‐83.4)	**<.001**
sR_aw_, kPa/s, age 3	1.1 (0.9‐1.2)	1.5 (1.3‐1.6)	**<.001**
sR_aw_, kPa/s, age 5	1.2 (1.0‐1.3)	1.3 (1.1‐1.4)	**<.001**
sR_aw_, kPa/s, age 8	1.2 (1.0‐1.3)	1.8 (1.5‐1.9)	**<.001**
Methacholine DRR slope, age 8	11.9 (4.9‐17.4)	14.9 (2.5‐21.3)	.08
FeNO, ppb, age 8	11.5 (7.8‐19.5)	58.5 (24.2‐79.3)	**<.001**

Bold values relate to statistically significant results.

*Note* : FEV_1_ = forced expiratory volume in 1 second, FeNO = fraction of exhaled nitrogen oxide; t test used for differences between means.

#### 3.2.2 Comparison with children who never wheezed, and wheezers with no severe exacerbations

Table E3 shows the results of the multinomial logistic regression comparing demographic data, early‐life risk factors, and co‐morbidities between children who wheezed, but have not had severe exacerbations (WNE), and those in the two severe exacerbation clusters (IE and FE), using children who have never wheezed (NW) as reference. The duration of breastfeeding was significantly shorter among the two exacerbation clusters. Maternal smoking during pregnancy significantly increased the risk of all 3 groups characterized by the presence of wheezing, with the highest magnitude of risk among children in the FE cluster. Rhinitis in mid‐childhood and early‐life eczema was associated with FE cluster.

Lung function data and FeNO in the four groups are shown in Table E4. Lung function was significantly diminished in all 3 groups of children who have wheezed, with significantly lower levels in the FE cluster. FeNO was significantly increased in both exacerbation clusters (but not in the WNE group) and was significantly higher at age 8 years among those with FE compared to all other groups.

### 3.3 Asthma‐related outcomes in adolescence

Asthma‐related outcomes at age 16 years are shown in Table 3. Children in FE cluster were significantly more likely to have current asthma in adolescence than those who wheezed, but did not have severe exacerbations in the first 8 years of life, or those in IF cluster (67% FE *vs.* 30% IE *vs.* 13% WNE, *P* < .001). Similarly, significantly higher proportion of children in FE cluster used ICS at age 16 years (77% FE *vs.* 15% IE *vs.* 18% WNE, *P* < .001). Children in the FE cluster were significantly more likely to have moderately severe asthma.

**Table 3:** Asthma‐related outcomes at age 16 years among children who wheezed, but have not had exacerbations (WNE), and children in the two exacerbation clusters (IE and FE)

	WNE (n = 244) N (%)	IE (n = 97) N (%)	FE (n = 9) N (%)	*P*‐value
Current asthma, age 16 years	32 (13%)	29 (30%)	6 (67%)	**.02**
Use of inhaled corticosteroids at age 16 years	45 (18%)	37 (15%)	7 (77%)	**.04**
Asthma severity				
No asthma treatment	202 (82.8%)	61 (62.8%)	1 (11.1%)	**<.001**
Step 1	24 (9.8%)	15 (15.6%)	1 (11.1%)	
Step 2	14 (5.8%)	14 (14.4%)	3 (33.3%)	
Step 3 or above	4 (1.6%)	7 (7.2%)	4 (44.4%)	

Bold values relate to statistically significant results.

* Fisher's exact test for asthma treatment due to small numbers. Chi‐squared used for binary data.

**Figure 2:**
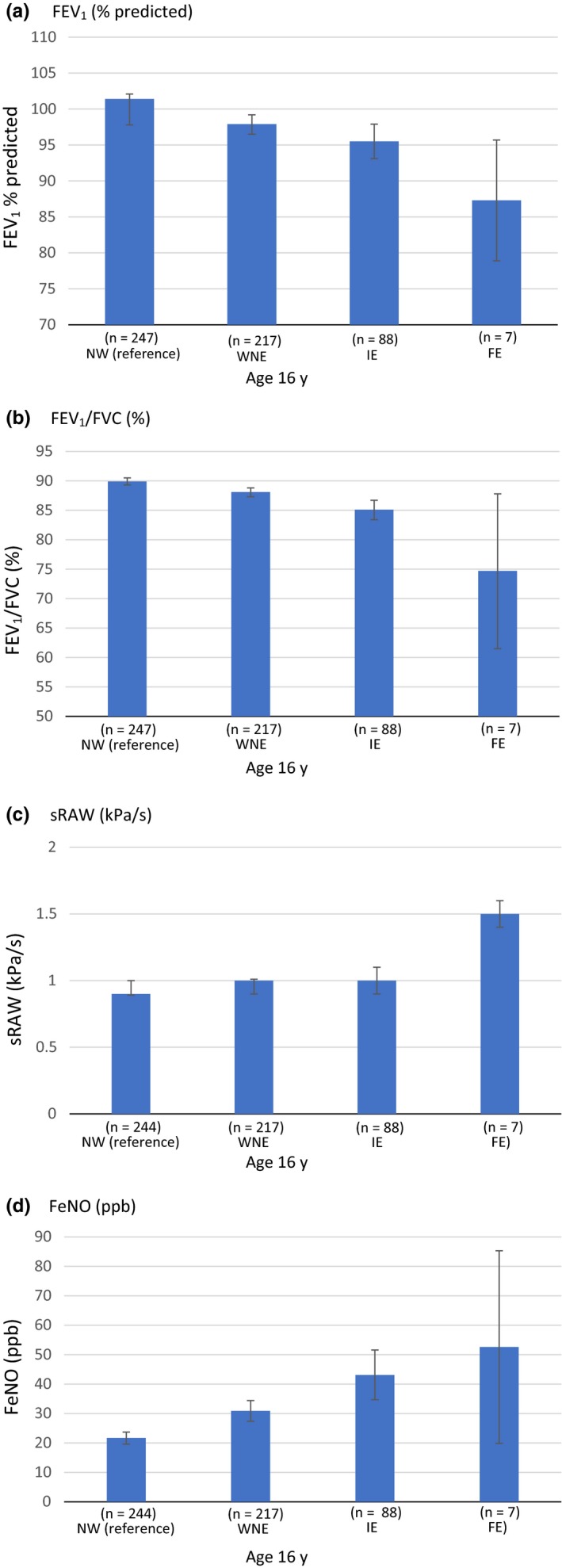
Lung function and FeNOat age 16 years among children who never wheezed (NW), those who wheezed, but have not had exacerbations (WNE), and children in the two exacerbation clusters (IE and FE). Children with lung function tests, N = 559. See online supplement for visualization. FEV_1_ = forced expiratory volume in 1 second, FeNO = fraction of exhaled nitrogen oxide. Quantitative variables represented as mean (95% confidence interval)

Lung function and FeNO at age 16 years are shown in Figure 2 and Table E5. Lung function (ascertained by FEV_1_% predicted, FEV_1_/FVC and sR_aw_) was significantly diminished in the FE cluster, indicating obstructive pattern (FEV_1_/FVC, mean [95%CI]: 89.9% [89.3‐90.5] *vs.* 88.1% [87.3‐88.8] *vs.* 85.1% [83.4‐86.7] *vs.* 74.7% [61.5‐87.8], NW, WNE, IE, FE, respectively, *P* < .001). FeNO levels were significantly higher in all three groups of children who have wheezed compared to NW, with the highest levels among those in the FE cluster.

**Figure 3:**
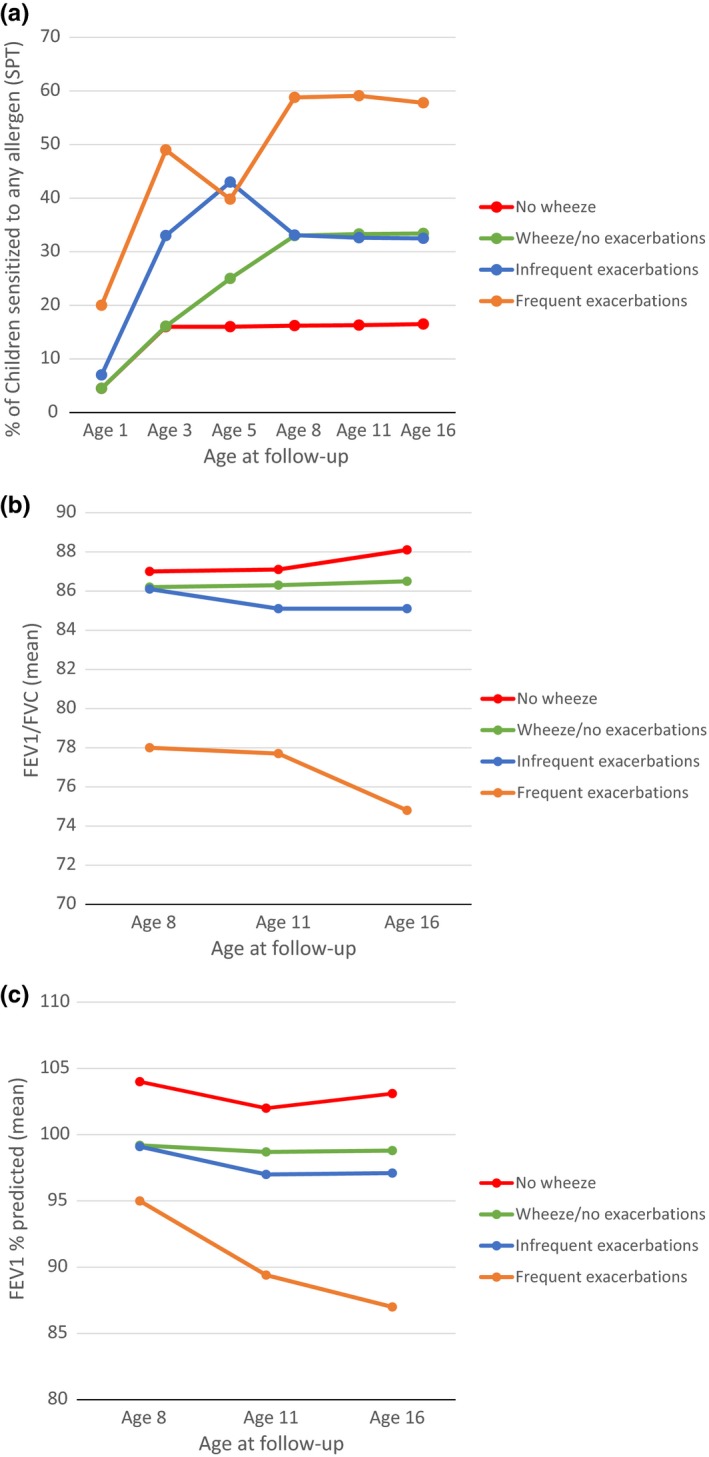
Longitudinal profiles of: (A) allergic sensitization; (B) FEV1/FVC; and (C) FEV1% predicted, among children who never wheezed, those who have wheezed but had no exacerbations, and the two exacerbation clusters

Figure 3 shows longitudinal profiles of allergic sensitization and lung function across the four groups. Sensitization started early, increased steadily, and remained higher throughout childhood in the FE cluster compared with other groups (Figure 3A). FEV_1_ and FEV_1_/FVC were significantly lower from mid‐school age to adolescence in the FE cluster, and declined from age 8 to age 16 years among children in this, but not in any other group (Figure 3B,C).

## 4 DISCUSSION

### 4.1 Key findings

To the best of our knowledge, this is the first study that has mapped longitudinal trajectories of severe of exacerbations of wheeze/asthma during childhood using data‐driven methodologies. We identified two distinct trajectories of severe exacerbations from birth to mid‐school age: Infrequent exacerbations and early‐onset frequent exacerbations. Both trajectories were associated with persistent wheeze, but had different early‐life risk factors, and different asthma‐related outcomes and lung function in adolescence. Shorter duration of breastfeeding was the strongest early‐life risk factor for the early‐onset frequent exacerbations. Lung function, measured from pre‐school age using sR_aw_, was significantly diminished in children in frequent compared with those in the infrequent severe exacerbations trajectory throughout childhood. By adolescence, children assigned to the frequent exacerbations trajectory were more likely to have a diagnosis of asthma and were more likely to use inhaled corticosteroids, compared with those in the infrequent exacerbations trajectory and children who have wheezed but had no severe exacerbations. Children in this trajectory also had significantly diminished lung function and greater airway inflammation in adolescence. Furthermore, lung function declined from age 8 to age 16 years among children in the early‐onset frequent exacerbations cluster, but not in any other group. Of note, we identified a subset of children who had frequent exacerbations, despite having mild asthma with apparently good symptom control and normal lung function.

### 4.2 Limitations and strengths

One limitation of our study is that we do not have a replication population, as another birth cohort with transcribed data from healthcare records to allow objective ascertainment of severe exacerbations could not be identified. Another limitation arises from the number of children included in the analyses to determine longitudinal trajectories. Given the relatively small number of children in the early‐onset frequent exacerbations trajectory, the study may not have picked up all differences compared with other groups, but only the highly significant ones. However, severe exacerbations are relatively rare events. Children who exacerbated made up 32% of our wheezing population, which is similar to other UK cohorts. Non‐UK cohorts showed either lower^3^, ^30^ or higher^31^ exacerbation rates, although some of the studies reporting higher exacerbation rates were carried out in high‐risk populations with preponderance of severe asthmatics.^31^

We acknowledge that there may be an underestimation of severe exacerbations among pre‐school children as a consequence of a relatively stringent definition we used, and we cannot exclude the possibility that the underestimation of severity may be more likely in the first year of life. Currently, there is no consensus on how to define severe wheeze exacerbations in pre‐school age. Our definition included hospital admissions and emergency department visits requiring the use of oral and/or injectable corticosteroids— that is the phenotype which we focussed on relates to genuinely severe exacerbations. Importantly, our definition is based on objective data derived from healthcare records, which contain precise information on physician‐confirmed episodes of wheeze, systemic corticosteroid prescriptions, emergency department admissions, and/or wheeze or asthma‐related hospitalizations. Age in days at the time of each event was recorded, and we did not have to rely on the recollection of parents, which has been shown to be relatively inaccurate.^32^ Another strength is that we used data‐driven approach which has removed any a priori assumptions.

### 4.3 Interpretation

This study builds upon our previously published work in a well‐defined cohort of patients with asthma diagnosis.^3^ Using similar methodology, we identified an exacerbation‐prone asthma subtype, independent of asthma severity or control, which is consistent with current findings in the different setting of a population‐based birth cohort.

Duration of breastfeeding was the most significant early‐life risk factor for the early‐onset frequent exacerbations trajectory, and children with the shortest duration of breastfeeding were more likely to experience exacerbations in early childhood. Our findings are consistent with results of the recent study by Ahmadizar *et al*, which has shown that breastfeeding is associated with a decreased risk of asthma exacerbations in later childhood.^33^ These results are consistent with findings that breastfeeding may have immunomodulatory and anti‐infective antimicrobial effects,^34^ thereby impacting upon the risk of exacerbations (which are likely mostly virus‐induced).^35^ Breast milk contains high levels of immunoglobulins, lactoferrin, cytokines and prebiotic structures that influence the development of immune system.^36^ Furthermore, it has been shown that breast milk can alter the composition of gut microbiome.^37^ However, further experimental studies are required to clarify underlying mechanisms.

Children in the early‐onset frequent exacerbations trajectory were more likely to have co‐morbid eczema in early life (but not later in childhood). This is consistent with our recent finding that across different developmental profiles of wheeze, eczema and rhinitis, *filaggrin* locus (a major genetic risk factor for early‐onset eczema) is differentially associated with eczema with co‐morbid wheeze and rhinitis.^38^ This trajectory appeared to be associated with early‐onset allergic sensitization. This is in concordance with an exacerbation cluster identified by the Trousseau Asthma Programme, which could suggest that similar risk factors may be contributing to early‐life sensitization and wheeze exacerbations.^39^

We have observed a relatively high frequency of rhinitis in early school age among all children with physician‐confirmed wheezing, with the proportion of subjects with rhinitis steadily increasing from those with no exacerbations (30%), to those in infrequent (37%) and frequent exacerbations trajectories (50%), compared to only 17% among children who have never wheezed. This extends recent findings that childhood rhinitis confers significant risk for asthma development^40^ and suggests that both allergic non‐allergic mechanisms are important for susceptibility to frequent exacerbations.^41^

The proportion of children with more severe asthma in our cohort was highest among children who had frequent exacerbations. However, a subset of children who experienced exacerbations had mild asthma with good symptom control, relatively low medication use and normal lung function. The could be explained in two ways: either these children were not on adequate therapy (eg only 50% of children in with FE trajectory were using ICS at age 8) and were therefore having exacerbations, or, that these children were on correct symptomatic therapy, but continued to exacerbate due to some other underlying mechanism which is not influenced by ICS treatment. Our data provide indirect evidence that exacerbation‐prone asthma may represent a separate asthma subtype, not necessarily associated with disease severity or control, or airway obstruction. Other recent studies using similar approaches have supported this notion.^3^, ^7^ These findings emphasize the need for further research into mechanisms underlying susceptibility to severe exacerbations.^42^

We found that frequent exacerbations trajectory was associated with more airway inflammation, airway hyperresponsiveness and poorer lung function, which is in line with previous studies.^4^, ^7^, ^31^ It is of note that FEV_1_/FVC ratio was considerably and significantly lower among children with frequent exacerbations, and that it declined during childhood. A recent multi‐cohort study has shown that frequent wheeze exacerbations in early childhood are associated with persistently low lung function trajectory which persists from early school age to the physiological peak in early adulthood,^43^ and increases the risk of the development of chronic obstructive pulmonary disease.^44^ Therefore, developing strategies to reduce early‐life severe exacerbations may contribute to the improvement in lifelong lung health.

In conclusion, we have identified two distinct patterns (trajectories) of severe exacerbations of wheezing during childhood with different early‐life risk factors and asthma‐related outcomes in adolescence. Our results provide a proof‐of‐concept that there are different exacerbation trajectories, and that these trajectories are likely associated with different mechanisms. However, the proportion of subjects with early‐onset frequent exacerbations is inevitably low (even in large birth cohort studies), rendering investigations into mechanisms difficult. Pooling the data from multiple birth cohorts, such as in STELAR^45^ and CREW^46^ consortia, may facilitate such studies (eg investigating genetic predictors of different exacerbation trajectories). However, to gain insight into the mechanisms, it is likely that any findings will have to be coupled with data generated in careful studies of patients with frequent exacerbations, and in mechanistic studies in human and animal models.^47^
