# Supplementary material for: Longitudinal trajectories of severe wheeze exacerbations from infancy to school age and their association with early‐life risk factors and late asthma outcomes
Source: Clin Exp Allergy. 2020 Jan 21;50(3):315–24. doi: 10.1111/cea.13553 (PMC7065181; doi:10.1111/cea.13553)
Supplement: Supplementary file 1 [file CEA-50-315-s001.docx]

**Longitudinal trajectories of severe wheeze exacerbations from infancy to school age and their association with early-life risk factors and late asthma outcomes**

Matea Deliu MD^1^, Sara Fontanella PhD^2^, Sadia Haider PhD^2^, Matthew Sperrin PhD^1^, Nophar Geifman PhD^1^, Clare Murray MD^3^, Angela Simpson MD PhD^3*^, Adnan Custovic MD PhD^2*^

*Joint senior authors

1. Division of Informatics, Imaging, and Data Science, Faculty of Medicine, Biology, and Health, University of Manchester, Manchester UK

2. National Heart and Lung Institute, Imperial College of Science, Technology, and Medicine, London UK

3. Division of Infection, Immunity, and Respiratory Medicine, School of Biological Sciences, University of Manchester, Manchester UK

**Supplementary Material**

**METHODS**

*Study design:* Unselected birth cohort

Setting: A mixed urban-rural population within 50 square miles of South Manchester and Cheshire, United Kingdom located within the maternity catchment area of Wythenshawe and Stepping Hill Hospitals

*Screening and recruitment*: All pregnant women were screened for eligibility at antenatal visits (8-10^th^ week of pregnancy). Of the 1499 couples who met the inclusion criteria (≤10 weeks of pregnancy, maternal age ≥18 years, and questionnaire and skin prick data test available for both parents), 288 declined to take part in the study and 27 were lost to follow-up between recruitment and the birth of a child. A total of 1184 children born into the study had at least some evaluable data.

*Data from primary care medical records:* Eligible GP practices were invited to participate in the study by postal information packs and telephone calls. Data access and manual extraction were performed during arranged visits to each GP practice. A trained paediatrician extracted data from electronic and paper-based primary care medical records, including prescriptions, acute wheeze episodes, hospital admissions for asthma/wheeze, oral steroid prescriptions. Timing, type of visit, symptoms, indication and prescriptions were noted for each encounter.

***Definition of variables***

*Wheeze phenotypes*^1,2^*:* (1) No wheezing: no wheeze ever; (2) Transient early wheezing: wheezing during the first 3 years, no wheezing in the previous 12 months at subsequent follow-ups; (3) Late-onset wheezing: no wheeze during the first 3 years, reported wheezing in the previous 12 months at age 5 years or later; and (4) Persistent wheezing: wheezing throughout childhood.

*Current rhinitis:* Positive answer to “In the past 12 months, has your child had a problem with sneezing or a runny or blocked nose when he/she did not have a cold or the flu?”

*Current eczema:* Positive answer to “Has your child had eczema within the past 12 months?”

*Atopic sensitisation:* Mean diameter (MWD) 3mm larger than the negative control to at least one allergen.

*Current wheeze:* physician confirmed wheeze as documented in primary care records available each year up to age 8. From ages 11-16, current wheeze is documented as a positive answer to the question “Has your child had wheezing or whistling in the chest in the last 12 months?”

*Asthma medication*: The use of inhaled corticosteroids and/or other asthma medication was recorded in primary care records.

**Statistical Analysis**

*Identification of Exacerbation clusters*

We used a k-means longitudinal model to ascertain the longitudinal trajectories of exacerbations in childhood. The KmL^4^ technique belongs to the class of partitional clustering. The main advantages of these methods are that no distributional assumptions within clusters are required, no assumptions regarding the shape of the trajectories are made, and they are independent from time-scaling.

Formally, consider a set *S* of *n* subjects and let $x_{it}$ be the value of variable $X$ measured for each subject $i$ at time $t$*.* The sequence $\text{x}_{i}=(x_{i1},x_{i2},\ldots,x_{it})$ is then called a *trajectory*. The aim of KmL is, then, to divide *S* into *g* homogeneous sub*-*groups. Several distance measures can be chosen to assign trajectories to clusters. We used the Manhattan distance, which is more robust to outliers^4^. The optimal number of clusters is assessed using the Calinski and Harabatz criterion, which evaluates cluster validity based on the average between- and within-cluster sum of squares. Using all time points from 1-8, two optimal models were identified: a 2-cluster solution and a 4-cluster solution, with a slight preference to a 2-cluster solution (Figure E1). We then collapsed the time points to 1-3-5-8 years in order to reduce the variability and to correspond to clinical follow up.

To test the validity of exacerbation classes, we created a separate dataset that included the entire population with complete data (n=887). We allocated children to 2 extra a priori identified subgroups which we identified as “children with no wheeze at baseline” and “children with wheeze but no exacerbations”. We compared early and late childhood risk factors and lung function between the exacerbation subgroups and the aforementioned a priori ones.

**Figure E1.** Calinski-Harabatz criterion for assessing optimal number of clusters.

Optimal solution

**Table E1.** Number of children with at least one exacerbation between birth and age 8 years and descriptive characteristics of children who ever had an exacerbation based on primary care records

ICS: inhaled corticosteroid

| **Age (years)** | | **0-1** | **1-2** | **2-3** | **3-4** | **4-5** | **5-6** | **6-7** | **7-8** |
| --- | --- | --- | --- | --- | --- | --- | --- | --- | --- |
| Number of children with at least one exacerbation in each year of life between birth and age 8 years, n= 887 | | 45/887, 5.1% | 44/887, 5.0% | 44/887, 5.0% | 54/887, 6.0% | 30/887, 3.4% | 20/887, 2.2% | 20/887, 2.2% | 14/887, 1.6% |
| Current ICS in current exacerbators, n=160 | | 14/45, 31% | 18/44, 41% | 20/44, 45% | 27/54, 50% | 20/30, 67% | 14/20, 70% | 10/20, 50% | 11/14, 79% |
| Current asthma medication in current exacerbators, n=160 | All exacerbations | 33/45, 73% | 36/44, 82% | 41/44, 93% | 47/54, 87% | 27/30, 90% | 19/20, 95% | 17/20, 85% | 12/14, 86% |
|  | ≥3 | 5/5, 100% | 6/7, 86% | 10, 100% | 1/1, 100% | 2/2, 100% | 1/1, 100% | 2/2, 100% | 1/1, 100% |
| Gender (boys), n=160 | | 108/160, 67% | | | | | | | |
| Ever asthma in ever exacerbators, n=160 | | 68/160, 43% | | | | | | | |
| Ever ICS in ever exacerbators, n=160 | | 116/160, 73% | | | | | | | |

**Table E2.** Distribution of asthma severity by exacerbation clusters.

IF: Infrequent exacerbations; FE: Early-onset frequent exacerbations; BTS: British Thoracic Society step in asthma treatment. Missing data assumed as missing at random. **Fisher’s exact test used due to low numbers. Bolded values represent significant p-values

|  | **Cluster 1 (IF)** | **Cluster 2 (FE)** | **p-value** |
| --- | --- | --- | --- |
| **BTS age 3y**** | **N=142** | **N=10** |  |
| No asthma treatment | 62 (43.7%) | 1 (10%) | **0.03** |
| Step 1 | 48 (33.8%) | 3 (30%) |  |
| Step 2 | 31 (21.8%) | 5 (50%) |  |
| Step 3 and above | 1 (1.7%) | 1 (10%) |  |
| **BTS age 5y**** | **N=135** | **N=10** |  |
| No asthma treatment | 55 (40.7%) | 1 (10%) | 0.19 |
| Step 1 | 33 (24.4%) | 3 (30%) |  |
| Step 2 | 40 (29.6%) | 5 (50%) |  |
| Step 3 and above | 7 (5.2%) | 1 (10%) |  |
| **BTS age 8y**** | **N=95** | **N=9** |  |
| No asthma treatment | 37 (39%) | 1 (10%) | 0.31 |
| Step 1 | 18 (19%) | 3 (30%) |  |
| Step 2 | 32 (34%) | 4 (40%) |  |
| Step 3 and above | 8 (8%) | 1 (10%) |  |

**Figure E2.** Trajectories of sRaw in from age 3 to age 8 years among children in two exacerbation trajectories

**Table E3.** Demographics, early-life risk factors, and co-morbidities among children who wheezed, but have not had severe exacerbations (WNE), and those in the two severe exacerbation clusters (IE and FE): Results from the multinomial logistic regression using children who never wheezed (NW) as the reference.

SPT: skin prick test; quantitative continuous variable presented as median (IQR); ordinal variables represented as frequencies (%); RR=relative risk; CI=confidence interval. Bold values represent significant p-values.

|  | **NW (reference) (n=389)** | **WNE (n=338)**  **RR (95%CI)**  **p-value** | **IE (n=150)**  **RR (95%CI)**  **p-value** | **FE (n=10)**  **RR (95%CI)**  **p-value** |
| --- | --- | --- | --- | --- |
| Gender (boys) | 162 (42%) | **166 (49%)**  **1.5 (1.1-2.0)**  **0.01** | **82 (55%)**  **2.5 (1.7-4.0)**  **<0.001** | 5 (50%)  1.2 (0.3-4.1)  0.81 |
| Family history of asthma | 91 (23%) | **121 (36%)**  **1.8 (1.3-2.4)**  **<0.001** | **51 (34%)**  **1.6 (1.1-2.2)**  **0.01** | 3 (30%)  1.2 (0.4-4.3)  0.74 |
| Younger sibling | 165 (42%) | 136 (40%)  0.91 (0.64-1.22)  0.54 | **48 (32%)**  **0.65 (0.43-0.98)**  **0.04** | 3 (30%)  0.76 (0.18-3.23)  0.71 |
| Older Sibling | 208 (53%) | 170 (50%)  0.88(0.65-1.2)  0.39 | **93 (62%)**  **1.5 (1.0-2.3)**  **0.03** | 5 (50%)  0.87 (0.25-3.1)  0.83 |
| Breastfeeding (weeks), median (IQR)* | 10 (0-28) | 8 (0-24)  0.99 (0.98-1.0)  0.08 | **6 (0-20)**  **0.98 (0.97-0.99)**  **0.006** | **0 (0-1.8)**  **0.92 (0.85-0.99)**  **0.009** |
| Day care attendance | 280 (72%) | 212 (63%)  0.76 (0.55-1.1)  0.11 | **78 (52%)**  **0.59 (0.39-0.91)**  **0.01** | 7 (70%)  1.3 (0.25-6.2)  0.77 |
| Maternal smoking during pregnancy | 101 (26%) | **125 (37%)**  **1.6 (1.2-1.9)**  **<0.001** | **53 (35%)**  **1.4 (1.1-1.9)**  **0.02** | **5 (50%)**  **2.8 (1.3-6.3)**  **0.01** |
| Tobacco exposure, age 1 year | 96 (25%) | **116 (34%)**  **1.6 (1.2-2.2)**  **0.004** | 46 (31%)  1.4 (0.9-2.1)  0.14 | 4 (40%)  2.0 (0.6-7.4)  0.28 |
| Tobacco exposure, age 3 years | 89 (23%) | **116 (34%)**  **1.9 (1.4-2.7)**  **<0.001** | **46 (31%)**  **1.8 (1.2-2.8)**  **0.005** | 4 (40%)  2.6 (0.7-9.9)  0.15 |
| Tobacco exposure age 5 years | 91 (23%) | **119 (35%)**  **1.8 (1.3-2.5)**  **<0.001** | 42 (28%)  1.3 (0.9-2.5)  0.19 | 5 (50%)  3.2 (0.9-11.3)  0.07 |
| Dog ownership, birth | 54 (14%) | 62 (18%)  1.4 (0.9-2.1)  0.09 | 20 (13%)  1.0 (0.6-1.8)  0.88 | 3 (30%)  3.1 (0.7-12.8)  0.12 |
| Cat ownership, birth | 72 (18%) | 76 (22%)  1.3 (0.9-1.9)  0.15 | 24 (16%)  0.9 (0.5-1.5)  0.70 | 4 (40%)  3.5 (0.9-13.4)  0.06 |
| Rhinitis, age 5 years | 65 (17%) | **103 (30%)**  **2.3 (1.6-3.2)**  **<0.001** | **55 (37%)**  **3.2 (2.0-4.9)**  **<0.001** | **5 (50%)**  **7.9 (1.8-34.2)**  **0.005** |
| Eczema, age 1 year | 119 (31%) | 122 (36%)  1.3 (0.9-1.8)  0.06 | **54 (36%)**  **1.6 (1.1-2.5)**  **0.02** | **7 (70%)**  **15.3 (1.9-126.2)**  **0.01** |
| Eczema, age 3 years | 78 (20%) | 81 (24%)  1.3 (0.9-1.9)  0.13 | **47 (31%)**  **2.3 (1.5-3.5)**  **<0.001** | **6 (60%)**  **11.5 (2.3-58.1)**  **0.004** |
| Eczema, age 5 years | 105 (27%) | 105 (31%)  1.2 (0.9-1.7)  0.26 | **56 (37%)**  **1.6 (1.1-2.5)**  **0.01** | 4 (40%)  2.6 (0.6-10.4)  0.19 |

**Table E4.** Associations of exacerbation clusters with lung function: multinomial logistic regression using children who never wheezed (NW) as the reference.

FEV_1_= forced expiratory volume in 1 second, FVC= forced vital capacity, FeNO= fraction of exhaled nitrogen oxide

|  | **NW (reference) (n=389)** | **WNE (n=338)**  **RR (95%CI)**  **p-value** | **IE (n=150)**  **RR (95%CI)**  **p-value** | **FE (n=10)**  **RR (95%CI)**  **p-value** |
| --- | --- | --- | --- | --- |
| FEV_1_ % predicted, mean (SD), age 8 years | 103.9 (11.9) | **98.9 (11.7)**  **0.97 (0.95-0.98)**  **<0.001** | **95.6 (14.6)**  **0.95 (0.03-0.97)**  **<0.001** | **91.1 (14.6)**  **0.93 (0.87-0.99)**  **0.02** |
| FEV_1_/FVC, mean (SD), age 8 years | 87.9 (5.2) | **86.3 (5.8)**  **0.94 (0.91-0.97)**  **0.001** | **85.1 (7.6)**  **0.91 (0.88-0.95)**  **<0.001** | **78.1 (6.9)**  **0.80 (0.71-0.89)**  **<0.001** |
| sR_aw_, mean (SD), age 3 years | 1.1 (0.2) | **1.2 (0.2)**  **2.6 (1.1-6.3)**  **0.04** | **1.2 (0.3)**  **12.5 (4.3-36.3)**  **<0.001** | **1.5 (0.3)**  **86.7 (1.1-743.2)**  **<0.001** |
| sR_aw_, mean (SD), age 5 years | 1.1 (0.2) | **1.2 (0.2)**  **5.3 (2.5-11.2)**  **<0.001** | **1.2 (0.2)**  **9.8 (4.1-23.7)**  **<0.001** | **1.3 (0.2)**  **77.8 (11.4-532)**  **<0.001** |
| sR_aw_, mean (SD), age 8 years | 1.1 (0.2) | **1.2 (0.2)**  **1.99 (1.07-3.73)**  **0.03** | **1.2 (0.3)**  **4.4 (2.0-9.3)**  **<0.001** | **1.8 (0.2)**  **62.5 (11.1-353.4)**  **<0.001** |
| FeNO, mean (SD), age 8 years | 9.4 (13.3) | 9.6 (20.3)  1.01 (0.99-1.03)  0.08 | **11.5 (19.5)**  **1.02 (1.00-1.03)**  **0.01** | **58.5 (24.2-79.3)**  **1.05 (1.02-1.08)**  **<0.001** |

**Table E5.** Lung function at age 16 years among children who never wheezed (NW), those who wheezed, but have not had exacerbations (WNE), and children in the two exacerbation clusters (IE and FE). Children with lung function tests, N=559.

FEV1= forced expiratory volume in 1 second, FeNO= fraction of exhaled nitrogen oxide. Quantitative variables represented as mean (95% confidence interval).

|  | **NW (reference)**  **(n=247)** | **WNE (n=217)** | **IE (n=88)** | **FE (n=7)** | **p-value** |
| --- | --- | --- | --- | --- | --- |
| FEV_1_ % predicted, mean (95%CI) | 101.4 (99.8-102.1) | 97.9 (96.5-99.2) | 95.5 (93.1-97.9) | 87.3 (78.9-95.7) | 0.64 |
| FEV_1_/FVC, mean (95%CI) | 89.9 % (89.3-90.5) | 88.1 % (87.3-88.8) | 85.1 % (83.4-86.7) | 74.7 % (61.5-87.8) | **0.001** |
| sR_aw_, mean (95%CI) | 0.9 (0.91-1.0) | 1.0 (0.9-1.01) | 1.0 (0.9-1.1) | 1.5 (1.4-1.6) | 0.09 |
| FeNO, ppb, mean (95%CI) | 21.7 (19.7-23.8) | 30.9 (27.4-34.4) | 43.1 (34.6-51.5) | 52.6 (19.9-85.3) | **0.01** |

**REFERENCES**

1. Martinez FD, Wright AL, Taussig LM, Holberg CJ, Halonen M, Morgan WJ. Asthma and wheezing in the first six years of life. The Group Health Medical Associates. *The New England journal of medicine* 1995; **332**(3): 133-8.

2. Lowe LA, Simpson A, Woodcock A, Morris J, Murray CS, Custovic A. Wheeze phenotypes and lung function in preschool children. *American journal of respiratory and critical care medicine* 2005; **171**(3): 231-7.

3. James DR, Lyttle MD. British guideline on the management of asthma: SIGN Clinical Guideline 141, 2014. *Arch Dis Child Educ Pract Ed* 2016; **101**(6): 319-22.

4. Genolini C, Falissard B. KmL: a package to cluster longitudinal data. *Comput Methods Programs Biomed* 2011; **104**(3): e112-21.
